# Discordance in causal terminology between scientific papers and subsequent press releases: chronic traumatic encephalopathy in the “age of spin”

**DOI:** 10.3389/fneur.2026.1882586

**Published:** 2026-07-08

**Authors:** Shawn R. Eagle, Natalie Sherry

**Affiliations:** Department of Neurological Surgery, University of Pittsburgh, Pittsburgh, PA, United States

**Keywords:** causal terms, chronic traumatic encephalopathy, CTE, media, press release

## Abstract

**Introduction:**

The concept of “spin,” or overstating the conclusions and/or significance of the research findings in a press release, has been a notable problem across science. Whether spin is a problem for scientific communication about chronic traumatic encephalopathy (CTE) studies is unknown.

**Methods:**

A search for the terms “chronic traumatic encephalopathy” or “CTE” was conducted on EurekAlert!, returning 61 usable press releases. We calculated a novel metric (“causal intensity score”) for each scientific abstract and its corresponding press release. We quantified “spin” as the difference between the causal intensity score of the abstract/press release.

**Results:**

Thirty-nine of the 61 (63.9%) press releases studied were more deterministic than the abstract, reflected by a discordance gap >0.0. Five releases were categorized as “severe spin,” with z-scores ≥1.5 standard deviations above the mean.

**Discussion:**

In this novel investigation, we found that press releases on CTE research studies were more deterministic on average than the studies themselves.

## Introduction

Chronic traumatic encephalopathy (CTE) is a neuropathological entity characterized by a unique pattern of tau deposition in those exposed to repetitive head impacts. CTE has captured the public’s attention since the publication of the first case in a former professional American football player in 2005 (1). Over the last 20 years, prevalence of CTE science in the popular media has risen exponentially leading to greater public awareness of the disease (2, 3). Significant disparities exist between which CTE studies get more media attention (2). Specifically, studies which report an association between exposure to repetitive head impacts and affirm higher odds of having CTE, receive nearly double the new stories than those who do not identify such an association (2). Possible reasons for this phenomenon include that a majority of CTE studies have focused on former professional American football players, which is the most popular sport in the United States. There are also known negativity biases in popular media which may explain this relationship (4, 5).

Popular media consumption is often shaped by institution-initiated press releases of newly published scientific findings. A significant disparity in the rate of issued press releases has also been observed for CTE research (6), which may inadvertently promote select perspectives within the field and distort public perception about the state of CTE science. Several studies have shown that athletes and their families obtain their information on CTE from popular media and their knowledge about CTE mechanisms and outcomes is incomplete (7–10). It is therefore important that press releases accurately reflect the scientific findings and frame the results in a balanced manner within the context of broader knowledge on the topic.

The concept of “spin,” or overstating the conclusions and/or significance of the research findings in a press release, has been a notable problem across science over the last decade (11–13). Of particular importance is the misrepresentation of causality, or a direct, deterministic link between exposure and an outcome. Causality is typically reserved for well-controlled evidence from a randomized clinical trial that clearly demonstrates a cause and effect (13), but observational studies often inappropriately make causal claims (a problem previously noted in CTE research) (14). Investigation of whether spin is a problem for scientific communication about CTE studies is warranted given the known disparities in how this information has historically been disseminated (6). Assessing the degree of interpretive spin is particularly important for providers tasked with educating the public on a novel, complex research area that is subject to extensive media coverage (3). The purpose of this study was to quantify the amount of spin between scientific publications on CTE and their associated press releases.

## Methods

**Figure 1:**
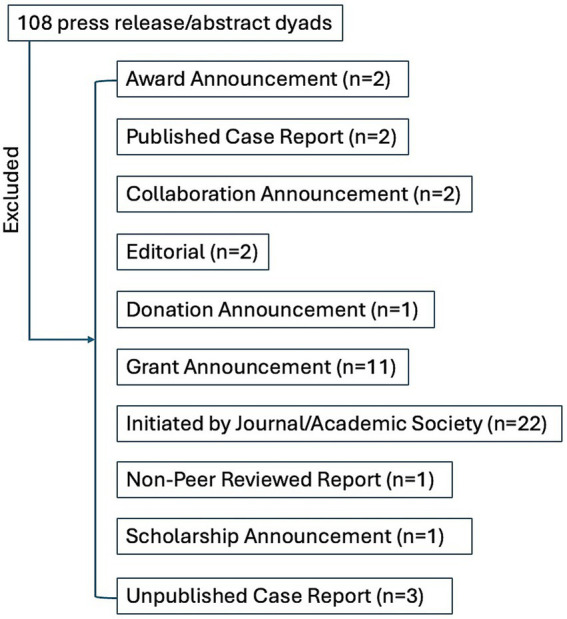
CONSORT diagram of included studies.

A search for the terms “chronic traumatic encephalopathy” or “CTE” was conducted on EurekAlert! on February 4th, 2026, returning 108 press releases. For the purposes of this investigation, we only included press releases issued from an academic institution about a peer-reviewed publication of original data or a review article. Examples of other topics for which press releases were issued (but not included in the current study) were those issued by a journal/academic organization, announcing receipt of grant funding, award recognition, non-peer reviewed autopsy findings, and case reports with no abstract. This resulted in a final analyzable sample of 61 press releases (Figure 1).

Each press release and the abstract from its corresponding scientific publication were extracted and compared. We operationalized the classification of causal claims using a lexical dictionary adapted from the manual coding frameworks of Sumner et al. (12) and Adams et al. (11). Terms were scored on an ordinal scale: Associative (0 points), Conditional (1 point), and Deterministic (2 points), consistent with the hierarchy of evidence strength described in the STROBE guidelines (15). A list of terms within each group can be viewed in Table 1. Reviewers were not blinded to the press release or abstract’s author list.

**Table 1:** Ordinal categories of causal terms derived from the manual coding frameworks of Sumner et al. (12) and Adams et al. (11).

	Deterministic/causal (2 points)	Conditional/suggestive (1 point)	Associative/safe (0 points)
Terms	cause, causes, caused, causing, cure, cures, cured, prevent, prevents, prevented, boost, boosts, boosted, reduce, reduces, reduced, increase, increases, increased, effect, effects, affect, affects, proven, proves, proof	imply, implies, suggest, suggests, predict, predicts, risk, marker, link, linked, relationship, relation	associated, correlation, correlated, related, observed, seen, measured

We calculated a novel metric termed the “causal intensity score” for each press release and a separate causal intensity score for the corresponding scientific abstract (i). This score was defined as the mean of all causal term point totals identified within each text, providing a measure of causal language severity. Texts containing no causal terms were assigned a score of 0.

- Causal Intensity Score = (Sum of Causal Term Points for Document)/ Total Number of Individual Causal Terms in the Document

We quantified “spin” (16) as the difference between the causal intensity score of the press release and its corresponding abstract (e.g., the “discordance gap” [ii]) (13). A positive discordance gap indicates that the press release utilized stronger causal language than the associated abstract.

- Discordance Gap = Press Release Causal Intensity Score – Abstract Causal Intensity Score

To identify patterns in the literature exhibiting significant deviations from the cohort norm, raw discordance gaps were standardized into z-scores (iii).

- *Z*-score = (Individual Discordance Gap – Mean Discordance Gap for All Press Release/Abstract Pairs)/ Standard Deviation of Discordance Gaps for All Press Release/Abstract Pairs

Observations were classified based on their deviation from the mean discordance of the entire sample:

- Severe spin: *Z*-score ≥ 1.5 standard deviations above the mean.
- Moderate spin: *Z*-score between 1.0 and 1.4 standard deviations above the mean.
- Accurate/normal range: *Z*-score within ±1.0 standard deviation.
- Cautious reporting: *Z*-score < −1.0 standard deviation (indicating the press release used weaker causal language than the abstract).

All data processing and statistical analyses were performed using R (version 4.5.2; RRID: SCR_001905) with the dplyr (version: 1.2.0; RRID: SCR_016708) and tidytext (version: 0.4.3; RRID: SCR_028593) packages.

## Results

**Figure 2:**
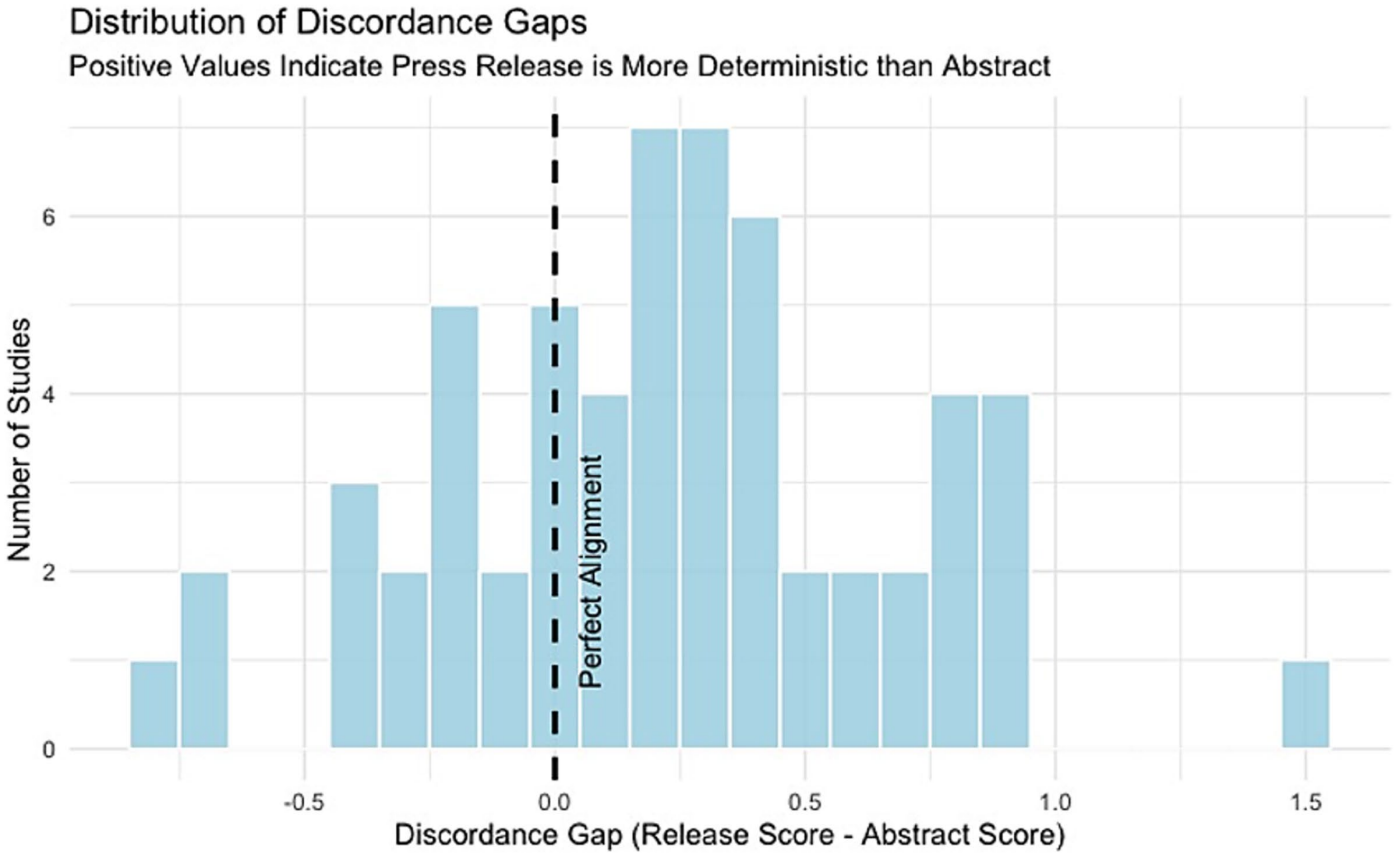
Distribution of discordance gaps, defined as the difference between the causal intensity score of the scientific abstract and its corresponding press release. Causal intensity scores were defined as the sum of causal term points for document divided by the total number of individual causal terms in the document.

Overall, 39 of the 61 (63.9%) press releases studied yielded a positive discordance gap indicating the press releases were more deterministic than the scientific abstract (Figure 2). The average discordance gap between abstract and press release was 0.225 with a standard deviation of 0.451. Two complete years had higher median discordance gaps than the group average: 2015 and 2019. A complete list of included studies with descriptive statistics can be viewed in Table 2. The average causal intensity score for abstracts was 0.740 and for releases was 0.964. The average number of causal terms identified in abstracts and releases was 6.9 and 10.1, respectively.

**Table 2:** List of press releases by institution and year, with causal intensity scores (CIS) for each abstract and press release, number of causal terms, used, discordance gap and “spin” verdict.

Institution	Year	Abstract CIS	Abstract causal terms	Release CIS	Release causal terms	Discordance gap	*Z*-score	Spin verdict
Indiana University	2019	0.50	2	2.0000000	1	1.50000000	2.8	Severe
Boston University	2026	0.80	5	1.7500000	12	0.95000000	1.6	Severe
Boston University	2024	0.60	5	1.5000000	4	0.90000000	1.5	Severe
Boston University	2018	0.25	4	1.1363636	22	0.88636364	1.5	Severe
Boston University	2024	0.00	7	0.8823529	17	0.88235294	1.5	Severe
University of Texas Health Science Center at San Antonio	2019	0.00	3	0.8333333	6	0.83333333	1.4	Moderate
Boston University	2023	0.50	4	1.3333333	6	0.83333333	1.4	Moderate
University of Western Ontario	2018	0.40	5	1.2000000	5	0.80000000	1.3	Moderate
University of California-Los Angeles	2015	0.33	3	1.1111111	18	0.77777778	1.2	Moderate
Boston University	2019	0.43	7	1.1250000	16	0.69642857	1.1	Moderate
University Health Network	2017	0.60	5	1.2727273	11	0.67272727	1.0	Accurate
Harvard Medical School	2020	1.00	4	1.6363636	11	0.63636364	0.9	Accurate
Boston University	2023	0.63	8	1.2121212	33	0.58712121	0.8	Accurate
Boston University	2019	0.20	5	0.7500000	4	0.55000000	0.7	Accurate
Mayo Clinic	2015	1.00	4	1.4545455	11	0.45454545	0.5	Accurate
Mass General Brigham	2023	0.50	4	0.9333333	15	0.43333333	0.5	Accurate
University College London	2017	1.00	5	1.4210526	19	0.42105263	0.4	Accurate
Boston University	2022	0.73	11	1.1428571	7	0.41558442	0.4	Accurate
Boston University	2023	0.92	13	1.3333333	9	0.41025641	0.4	Accurate
Ben-Gurion University of the Negev	2020	0.92	12	1.3076923	13	0.39102564	0.4	Accurate
Boston University	2025	0.91	21	1.2857143	14	0.38095238	0.4	Accurate
New York Medical College	2012	0.75	8	1.0909091	11	0.34090909	0.3	Accurate
Boston University	2022	0.29	7	0.6000000	5	0.31428571	0.2	Accurate
Boston University	2018	0.85	13	1.1538462	13	0.30769231	0.2	Accurate
Boston University	2023	0.20	20	0.5000000	10	0.30000000	0.2	Accurate
Boston University	2024	1.12	17	1.4000000	10	0.28235294	0.1	Accurate
NYU Langone Health	2025	0.86	14	1.1333333	15	0.27619048	0.1	Accurate
Boston University	2022	0.80	5	1.0714286	14	0.27142857	0.1	Accurate
Boston University	2021	0.25	8	0.5000000	6	0.25000000	0.1	Accurate
Boston University	2015	0.00	1	0.2500000	4	0.25000000	0.1	Accurate
Beth Israel Deaconess Medical Center	2015	1.25	4	1.4782609	23	0.22826087	0.0	Accurate
Mass General Brigham	2024	0.67	6	0.8461538	13	0.17948718	−0.1	Accurate
Boston University	2018	0.73	11	0.9047619	21	0.17748918	−0.1	Accurate
Boston University	2021	1.00	3	1.1666667	6	0.16666667	−0.1	Accurate
Boston University	2023	0.78	9	0.9375000	16	0.15972222	−0.1	Accurate
Boston University	2024	0.50	8	0.6250000	8	0.12500000	−0.2	Accurate
Northwestern University	2025	0.67	3	0.7777778	9	0.11111111	−0.3	Accurate
Boston University	2020	0.72	7	0.8000000	10	0.08571429	−0.3	Accurate
Boston University	2024	0.50	8	0.5833333	12	0.08333333	−0.3	Accurate
Mount Sinai Hospital	2025	1.00	2	1.0000000	6	0.00000000	−0.5	Accurate
Boston University	2021	0.50	2	0.5000000	2	0.00000000	−0.5	Accurate
University of Colorado at Boulder	2021	0.00		0.0000000	4	0.00000000	−0.5	Accurate
Boston University	2020	0.78	9	0.7777778	18	0.00000000	−0.5	Accurate
University of California-Los Angeles	2016	0.67	3	0.6666667	3	0.00000000	−0.5	Accurate
Boston University	2016	1.42	12	1.3333333	6	−0.08333333	−0.7	Accurate
Boston University	2023	0.69	16	0.5555556	9	−0.13194444	−0.8	Accurate
Boston University	2022	1.40	10	1.2500000	8	−0.15000000	−0.8	Accurate
Boston University	2017	0.91	11	0.7500000	4	−0.15909091	−0.9	Accurate
University of Pittsburgh	2023	0.67	6	0.5000000	6	−0.16666667	−0.9	Accurate
Boston University	2021	0.67	3	0.5000000	2	−0.16666667	−0.9	Accurate
University of California-Los Angeles	2018	0.50	2	0.2857143	7	−0.21428571	−1.0	Accurate
Boston University	2018	1.25	8	1.0000000	11	−0.25000000	−1.1	Cautious
Boston University	2023	1.75	4	1.4285714	7	−0.32142857	−1.2	Cautious
Boston Children’s Hospital	2025	1.30	10	0.8750000	8	−0.42500000	−1.4	Cautious
Boston University	2017	1.00	3	0.5714286	7	−0.42857143	−1.5	Cautious
Mount Sinai Hospital	2024	1.00	1	0.5625000	16	−0.43750000	−1.5	Cautious
Boston University	2021	1.33	3	0.6666667	3	−0.66666667	−2.0	Cautious
Boston University	2024	1.67	3	1.0000000	2	−0.66666667	−2.0	Cautious
Boston University	2012	1.00	3	0.2000000	5	−0.80000000	−2.3	Cautious

**Figure 3:**
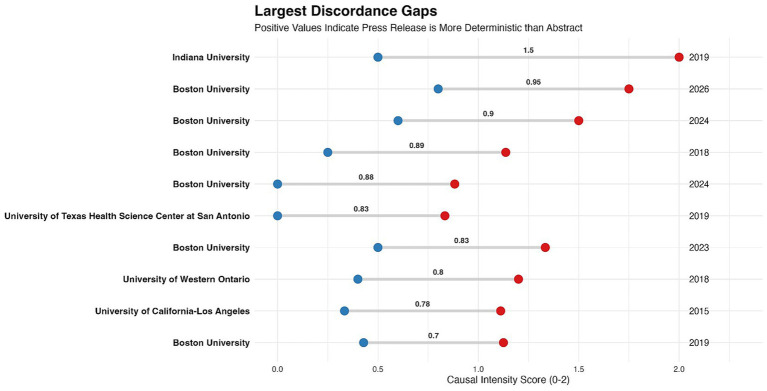
Ten largest discordance gaps of causal intensity scores between scientific abstract and corresponding press release. Causal intensity scores were defined as the sum of causal term points for document divided by the total number of individual causal terms in the document.

The 10 highest discordance gaps are shown in Figure 3. Overall, discordance gaps varied greatly across areas of scholarship related to CTE. Five releases were categorized as “severe spin,” with z-scores at least 1.5 standard deviations above the mean. Five releases were categorized as “moderate spin,” with z-scores between 1.0–1.4 standard deviations above the mean. Eight releases were categorized as “cautious,” with z-scores at least 1 standard deviation below the mean.

## Discussion

In this novel investigation on the dissemination of scientific findings related to CTE research, we found that press releases on CTE research studies were more deterministic on average than the studies themselves. This suggests that CTE press releases tend to assert stronger claims from research findings than what is considered acceptable by experts in the field. We identified several significant outliers which either directly or indirectly inferred causality in the press release, despite such conclusions not supported by the original research. The scientific community considers asserting a causal relationship between repetitive head impact exposure and CTE risk as premature and unsubstantiated by current evidence (14), thus making unsupported causal claims about repetitive head impact exposure and CTE risk can lead to widespread misinformation (3).

The oversaturation of negative media attention on a complex and nuanced topic like CTE could potentially influence underinformed, at-risk populations to make impulsive decisions about their own health. Former professional football players who assume they have CTE (despite that there are no validated *in vivo* diagnostic assessments) are much more likely to report suicidality and depression than players who do not assume they have CTE (17). Further, despite no difference in suicide rate for former National Football League (NFL) players from 1979–2019 to former professional baseball and basketball players, completed suicides in former football players increased two-fold after 2011 (18). The first documented press release about a CTE finding occurred in 2009 (6). To assume that CTE causes violent and/or suicidal behavior is an oversimplification of a complicated problem (3). Several studies have shown that former professional football players who have either been incorrectly diagnosed with CTE *in vivo* or meet criteria for traumatic encephalopathy syndrome (TES) have higher odds of co-reporting other treatable medical conditions that could account for their current symptoms (e.g., depression, anxiety, obstructive sleep apnea, low testosterone, headache disorder, etc.) (19, 20). The nonspecific nature of symptoms attributed to TES/CTE (21) raises the possibility of symptom misattribution (22). Individuals may be more likely to ascribe symptoms to an external cause, such as repetitive head injury, rather than to psychological or intrinsic factors, potentially due to stigma (23) and “good old days” bias which could impact their decision to seek treatment (24).

It is not known whether CTE messaging in the media causes individual despair or influences mental health. There are empirical examples, however, of media attention directly influencing individual patient’s decisions about their own medical care. A brief period of intense media coverage about side effects of statins caused 11–12% of patients with cardiovascular disease to cease taking the drug, often without consulting their physician (25). Another particular concern for CTE messaging is the Werther effect, which is a phenomenon of increased suicide rates following sensationalized depictions of a suicide (especially of a celebrity or public figure), often accompanied with oversimplifications related to cause of death (26). Several early CTE cases of high-profile professional football players who committed suicide were covered intensely in the media which could theoretically have induced copycat behaviors. It is the responsibility of scientists conducting this research to clearly and accurately communicate their findings to the public, to combat these potential risks.

## Conclusion

“Spin” of chronic traumatic encephalopathy research findings is a significant concern for many studies with associated press releases. Scientists are responsible for ensuring proper communication of research findings to the public to stop the spread of misinformation.

## Data Availability

The raw data supporting the conclusions of this article will be made available by the authors, without undue reservation.
